# A multi-protease, multi-dissociation, bottom-up-to-top-down proteomic view of the *Loxosceles intermedia* venom

**DOI:** 10.1038/sdata.2017.90

**Published:** 2017-07-11

**Authors:** Dilza Trevisan-Silva, Aline V. Bednaski, Juliana S.G. Fischer, Silvio S. Veiga, Nuno Bandeira, Adrian Guthals, Fabricio K. Marchini, Felipe V. Leprevost, Valmir C. Barbosa, Andrea Senff-Ribeiro, Paulo C. Carvalho

**Affiliations:** 1 Department of Cell Biology, Federal University of Paraná, Curitiba 81531-980, Brazil; 2 Computational Mass Spectrometry & Proteomics Group, Carlos Chagas Institute, Fiocruz, Curitiba 81.350-010, Brazil; 3 Center for Computational Mass Spectrometry, University of California, San Diego 92093-0404, USA; 4 Functional Genomics Laboratory, Carlos Chagas Institute, Fiocruz, Curitiba 81.350-010, Brazil; 5 Mass Spectrometry Facility RPT02H, Carlos Chagas Institute, Fiocruz, Curitiba 81.350-010, Brazil; 6 Systems Engineering and Computer Science Program, COPPE, Federal University of Rio de Janeiro, Rio de Janeiro 21941-914, Brazil; 7 Laboratory of Toxinology, Fiocruz, Rio de Janeiro 21040-900, Brazil

**Keywords:** Proteomics, Protein analysis, Entomology

## Abstract

Venoms are a rich source for the discovery of molecules with biotechnological applications, but their analysis is challenging even for state-of-the-art proteomics. Here we report on a large-scale proteomic assessment of the venom of *Loxosceles intermedia*, the so-called brown spider. Venom was extracted from 200 spiders and fractioned into two aliquots relative to a 10 kDa cutoff mass. Each of these was further fractioned and digested with trypsin (4 h), trypsin (18 h), pepsin (18 h), and chymotrypsin (18 h), then analyzed by MudPIT on an LTQ-Orbitrap XL ETD mass spectrometer fragmenting precursors by CID, HCD, and ETD. Aliquots of undigested samples were also analyzed. Our experimental design allowed us to apply spectral networks, thus enabling us to obtain meta-contig assemblies, and consequently *de novo* sequencing of practically complete proteins, culminating in a deep proteome assessment of the venom. Data are available via ProteomeXchange, with identifier PXD005523.

## Background & Summary

Scientists have long enlisted venoms in their quest to characterize novel molecules with biotechnological applications^1,2^. The literature provides innumerous examples of venom-derived applications, ranging from biopesticides to medical applications. In particular, works on serpent venom are, unarguably, success stories. Some examples are: Batroxobin, a widely used thrombin-like enzyme and commonly extracted from the venom of *Bothrops atrox* and *Bothrops moojeni*, has been used as a replacement for thrombin in bleeding injuries^3^; Ecarin, from *Echis carinatus*, as the primary reagent for laboratorial tests that monitor anticoagulation^4^; and Captopril, developed from peptides of the *Bothrops jararaca* venom, as a widely adopted inhibitor of the angiotensin converting enzyme (ACE). Other examples of venom-derived drugs include: Aggrastat, for myocardial infarct and ischemia; Ancrod, for stroke; Defibrase, for acute cerebral infarction and angina pectoris; Exanta, used as an anti-coagulant; Hemocoagulase, for hemorrhage; and Integrilin, for acute coronary syndrome^5^. Venoms have also been used to search for inhibitors derived from other species (e.g., *Didelphis marsupialis*)^6,7^.

Motivated by all the successful research on snake venoms, efforts have been geared towards spider toxins. In particular, those from the *Loxosceles* genus are already being used in at least four general application fronts, viz.: as therapeutic anti-venom sera^8^; as tools in molecular and cellular biology research; and as aids in drug development and production of selective and environmentally friendly bioinsecticides^5^. Peptides originating from the venom of *Thrixopelma pruriens* have been used in the treatment of pain and inflammation^9^; the T×2–5 and T×2–6 neuropeptides from the *Phoneutria nigriventer* venom, for treating erectile dysfunctions^10^; and distinct bioactive peptides from spider venoms, in the treatment of diverse diseases, such as cancer^11^. Taken together, toxins have served as an endless treasure trove for biotechnological applications.

Spider venoms, in particular, comprising mainly proteins and peptides^2,5,12,13^ and displaying great diversity in their toxins, have drawn considerable attention. Yet, characterizing venoms poses great challenges even for state-of-the-art proteomic strategies: in fact, most species lack a reference sequence genome^14^ and the post-translational modifications of venoms vary greatly. Moreover, current mainstream strategies are not tailored towards performing *de novo* sequencing of the large (i.e., greater than tryptic), biologically active peptides that abound in venoms. Indeed, peptide-centric approaches are oblivious to whether a sequenced peptide originates from a larger peptide or a full protein, but obtaining the complete sequence of these larger molecules will undoubtedly fuel a great diversity of biotechnological applications. In this regard, it is our view that widely adopted proteomic strategies such as peptide spectrum matching (PSM)^15,16^ and mainstream *de novo* sequencing^17^ only reveal the tip of the iceberg in terms of what can be unveiled from venoms.

One of our goals has been to characterize the venom of the so-called brown spiders (the *Loxosceles* genus). Altogether, their venom is composed of a complex cocktail of biologically active compounds, with toxins ranging up to 40 kDa and over^18^. To the best of our knowledge, an in-depth, comprehensive proteomic profiling of the *Loxosceles* venom tailored towards the discovery of new molecules has so far remained elusive. Currently, there are several descriptions of enzymatic and non-enzymatic proteins from distinct *Loxosceles* species^19,20^. In 2003, a study aimed to investigate whether venoms of phylogenetically-related groups of *Haplogyne* spiders possess sphingomyelinase-D (SMD) toxins^21^. The study included 10 *Loxosceles* species and 2 *Sicarius* species, among other spider genera. The Amplex Red Phospholipase-D assay kit indicated SMD activity and these results were further supported by a Surface-Enhanced Laser Desorption/Ionization (SELDI) Time-of-Flight (TOF) analysis showing mass spectral peaks with m/z’s corresponding to those of SMD. *Loxosceles* SMDs, later referred to as phospholipases-D (PLDs), are known to be the major component of *Loxosceles* venoms and are the most well characterized toxin family in brown spider venoms. In 2005, two-dimensional protein profiles of the *L. intermedia*, *L. laeta*, and *L. gaucho* venoms were determined, but protein identification was focused only on the SMD toxins of the *L. gaucho* venom^22^. The identification of seven spots of interest was first attempted using data from Matrix-Assisted Laser Desorption/Ionization (MALDI) Time-of-Flight (TOF) Mass Spectrometry (MS) and Electrospray Ionization (ESI) quadrupole-time-of-flight Tandem Mass Spectrometry (MS/MS) for direct search of raw data using MASCOT^22^. Since the searches retrieved no significant match, *de novo* sequencing was performed and the resulting sequences were BLASTed against the non-redundant sequences, allowing SMD identification for all analyzed spots^22^. Only in 2009 was a proteomic study described that targeted the total protein content of the *Loxosceles* venom^23^. Although the *L. intermedia* venom was analyzed using Multi-Dimensional Protein Identification Technology (MudPIT)^24^, only 39 proteins were identified. Of these proteins, only 14 were described as toxins generally found in animal venoms^23^. Thus, this proteomic study seems to have severely underestimated the great toxin diversity of the *Loxosceles* venom, particularly in comparison to the many publications that already described distinct molecular clones from venoms of different *Loxosceles* species^25–32^. Transcriptome analyses of the *L. laeta* and *L. intermedia* venoms revealed a huge complexity of brown-spider venoms^19,20^. Specifically, the analysis of the *L. intermedia* venom described three classes of toxins comprising most toxin-encoding transcripts, such as peptides of low molecular mass (55.9%), astacin-like proteases (22.6%), and PLDs (20.2%). Also, transcripts similar to hyaluronidases, serine proteases, serine protease inhibitors, venom allergens, and members of the translationally controlled tumor protein (TCTP) family presented low levels of expression^20^. Although considerable information is now available on venom gland transcripts of *L. intermedia*, the total protein content of this venom has remained unclear. A previous study from our group applied two-dimensional immunoblots and zymograms on the venom of *L. intermedia*, *L. laeta*, and *L. gaucho*, and revealed several spots with differential volume containing proteins having gelatinolytic activity corresponding to astacin-like proteases^33^. These results corroborate that venoms from these species present a broad astacin-like family with many isoforms^22,33,34^.

The lack of genomic data from this arachnid prevents employing the PSM approach in full, so most of the weightlifting must be accomplished through *de novo* sequencing. Mainstream *de novo* sequencing, however, cannot efficiently handle unanticipated post-translational modifications, being far more prone to generating sequencing errors. This is because various molecules fail to provide enough mass spectral peaks during fragmentation to enable the sequencing of full peptides. To overcome these limitations, our dataset was acquired with multiple dissociation strategies applied to the same precursor (e.g., collision-induced dissociation (CID), higher-energy collisional dissociation (HCD), and electron-transfer dissociation (ETD)), thereby enabling the use of state-of-the-art *de novo* sequencing algorithms. These capitalize on complementary dissociation information and thus achieve unprecedented sequencing accuracy^35,36^. The use of different proteolytic enzymes on the venom aliquots unlocks the application of another very powerful paradigm, that of spectral networks^37,38^. These ‘specnets’ align spectra against one another, ultimately allowing the detection of unanticipated post-translational modifications. Moreover, they can assemble consensus mass spectra from overlapping peptides yielded by different proteolytic digests. A consensus spectrum thus obtained presents a better signal-to-noise ratio and allows for the *de novo* sequencing of amino-acid stretches far longer than those handled by the conventional approach. Once high-confidence *de novo* data are available, it becomes possible to employ tools, such as PepExplorer^39^ or Meta-SPS^37^, that apply pattern recognition approaches to the mapping of *de novo* sequencing data against sequences from homologous organisms, thereby facilitating biological interpretation.

By themselves, the meta-contig assemblies provided by spectral networks are not enough for one to conclude whether a biomolecule obtained 100% coverage. To pave the way in this direction, top-down proteomic data in combination with MS3 (i.e., product ion(s) selected from an MS/MS spectrum further fragmented and producing another tandem mass spectrum) and ETD were also acquired for a partition of the venom molecules into two sets (<~10 kDa and >~10 kDa). The top-down strategy consists of injecting intact proteins into the mass spectrometer, thus doing away with the inference limitations of the peptide-centric approach^40^. This provides complementary information to that of the networks and helps in the discovery of how much is required for obtaining full coverage. We anticipate that these data will be fundamental in the development of next-generation algorithms capable of bridging the gap between bottom-up, middle-down, and top-down proteomics.

Here, we present the first multi-protease, multi-dissociation, bottom-up-to-top-down proteomic dataset of the venom of *L. intermedia,* the ‘urban’ spider species commonly found in the city of Curitiba, Brazil^41^, along with an analysis using state-of-the-art tools. The approach stems from the motivation that multiple enzyme digestion increases protein coverage^42^, besides relying on different activation and acquisition methods.

## Methods

### Sample preparation

Adult *L. intermedia* specimens (both male and female) were collected in the wild in accordance with the Brazilian Federal System for Authorization and Information on Biodiversity (SISBIO-ICMBIO, license number 29801-1). Venom from 200 spiders was extracted through the electrostimulation method^43^ and immediately diluted in ammonium bicarbonate buffer 0.4 M/urea 8 M. Protein concentration was determined through the Coomassie blue method, using bovine serum albumin (BSA) as standard curve^44^. First, the venom was separated into two fractions using an ultra-filter unit (MW cutoff 10 kDa) (Millipore), one fraction containing venom proteins above ~10 kDa (400 μg) and the other containing venom proteins and peptides bellow ~10 kDa (90 μg). All procedures described next were performed equally for each fraction, after further dividing it into four aliquots, each of which was reduced with dithiothreitol (DTT) to a final concentration of 25 mM for 3 h at room temperature. Afterwards, the samples were alkylated with iodacetamide (IAA) to a final concentration of 80 mM for 15 min at room temperature in the dark. Each aliquot was digested with one of the follow enzymes: trypsin (Trypsin Gold, Mass Spectrometry Grade, Promega Corporation, Madison, cat. No. V5280, WI, USA), chymotrypsin (Promega, cat. No. V1062), and pepsin (Promega, cat. No. V1959) at the ratio of 1:50 (E:S). We note that an additional aliquot was stored and not digested. Three aliquots were incubated individually with each enzyme for 18 h, at 25 °C for chymotrypsin and 37 °C for trypsin and pepsin. The other aliquot was incubated for only 4 h with trypsin at 37 °C. Each digested fraction was divided into three aliquots and desalted with ultra-micro C-18 spin columns according to the manufacturer’s instructions (Harvard Apparatus). One of these three aliquots was stored for future use, another had its peptides desalted and directly submitted to reverse phase chromatography coupled online with an Orbitrap XL mass spectrometer. The third aliquot of the desalted peptides was eluted with 70% acetonitrile (ACN) and 0.1% formic acid, then dried in a speed vacuum concentrator, suspending buffer C (i.e., 10 mM of K_2_HPO_4_, 25%ACN, pH=3.0). Afterwards, the sample was passed through a micro strong cation exchanged spin column (SCX) according to the manufacturer’s instructions (Harvard Apparatus). Briefly, the column was equilibrated with buffer C, centrifuged for 1 min at 100×g, and the sample was eluted from the SCX spin column with increasing concentration of KCl, i.e., 100, 170, 290, and 400 mM. Finally, each fraction was desalted once more with ultra-micro C-18 spin columns according to the manufacturer’s instructions (Harvard Apparatus). All columns were then washed ten times with 0.1% formic acid and the peptides were eluted with buffer B (i.e., 70% acetonitrile, 0.1% formic acid) to proceed to next step.

### Mass spectrometry analysis

Each fraction of peptides, including the non-fractionated as well as those from the SCX fractionation, was previously desalted and subjected to an LC-MS/MS analysis on a nano-LC 1D plus System (Eksigent, Dublin, CA), an ultra-high performance liquid chromatography (UHPLC) system coupled with an LTQ-Orbitrap XL ETD (Thermo, San Jose, CA) mass spectrometer, at the Mass Spectrometry Facility RPT02H of the Carlos Chagas Institute (Fiocruz, Brazil). In these analyses, the peptide mixtures were loaded onto a column (75 mm i.d., 15 cm long), packed in-house with a 3.2 μm ReproSil-Pur C18-AQ resin (Dr Maisch) with a flow of 500 nl/min and subsequently eluted with a flow of 250 nl/min from 5 to 40% ACN in 0.5% formic acid in a 120 min gradient. The mass spectrometer was set to data-dependent mode to automatically switch between MS and MS/MS acquisition. Full-scan MS spectra (m/z 350–1,800) were acquired in the Orbitrap analyzer with resolution R=60,000 at m/z 400 (after accumulation to a target value of 1,000,000 in the linear trap) using survey mode. The three most intense ions were sequentially isolated and fragmented using CID, HCD, and ETD for the same precursor. Previous target ions selected for MS/MS were dynamically excluded for 60 s. The total cycle time was approximately 5 s. The general mass spectrometric conditions were: spray voltage, 2.4 kV; no sheath or auxiliary gas flow; ion transfer tube temperature, 100 °C; collision gas pressure, 1.3 mTorr; normalized collision energy using wide-band activation mode; 35% for MS/MS. Ion selection thresholds were of 5,000 counts for MS/MS. The parameters for each fragmentation type in MS/MS acquisitions were as follows. For CID: isolation width, m/z 2.5; normalized collision energy, 35; activation, q=0.25; activation time, 30 ms. For HCD: isolation width, m/z 2.5; normalized collision energy, 35; activation time, 30 ms; full width at half maximum resolution, 15,000. For ETD: isolation width, m/z 2.5; activation time, 100 ms.

### Bioinformatics analysis

The *de novo* sequencing approach employed in this work utilized multiple MS/MS spectra from overlapping peptides, generated from multiple proteases and of precursors analyzed with CID, HCD, and ETD spectrum triples. Each was then converted into prefix residue mass (PRM) spectra. In this conversion, MS/MS peak masses were converted into putative cumulative precursor fragment masses, with intensity scores determined from likelihood models specific to each fragmentation mode. Triples of PRM spectra from the same precursor were then merged into a single PRM spectrum per precursor by adding scores for matching peak masses. Spectral-network algorithms, implemented in the ProteoSAFe web platform that is freely accessible at http://proteomics.ucsd.edu/ProteoSAFe/, were then used to align merged PRM spectra from peptides with overlapping sequences. Moreover, A-Bruijn algorithms were used to integrate these alignments into assembled contigs.

Each contig was then used to construct a consensus contig spectrum, or meta-contig, capitalizing on the corroborating evidence from all of its assembled spectra to yield a high-quality consensus *de novo* sequence^36^. Subsequently, the Meta-SPS algorithm was used to align the meta-contigs against a FASTA sequence database^37^. This database contained all *Loxosceles* sequences from UniProt, all from the transcriptome of the *L. intermedia* venom gland^20^, and an internal database with common mass spectrometry contaminants and proteases.

**Figure 1:**
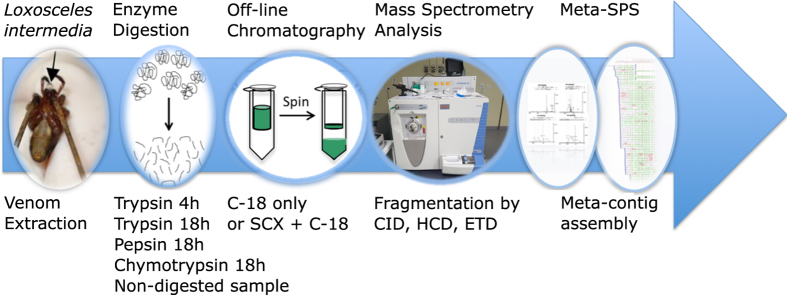
Methodology workflow. Summary of the sequence of procedures that constitute the methodology employed, from venom extraction to the meta-contig assembling that enabled the identifications of venom proteins.

A summary of this methodology is found in Fig. 1.

## Data Records

Our bioinformatics analysis disclosed a list of 190 proteins (Table 1). As far as we know, this is the most complete comprehensive proteomic profiling of the *L. intermedia* venom. All mass spectrometry data are available from both the ProteomeXchange Consortium via the PRIDE^45^ partner repository, with dataset identifier PXD005523 (Data Citation 1), and our servers (http://proteomics.fiocruz.br/pcarvalho/lintermedia/venom/). A full list of the proteins, meta-contigs, and homologous sequences is made available in Table 1.

All Meta-SPS results for >~10 kDa and <~10 kDa, together with the parameter files used for running the software, are available as separate material (MetaSPS_Results.xlsx, Data Citation 2). The results are presented in six tabs, viz., for >~10 kDa grouped by contig, >~10 kDa grouped by spectrum, >~10 kDa parameter file, <~10 kDa grouped by contig, <~10 kDa grouped by spectrum, and <~10 kDa parameter file.

## Technical Validation

The lack of any previous comprehensive proteomic analysis of the *Loxosceles* venom demonstrates that studying this venom in detail has been a challenge, one that stems from the organism being highly non-canonical and from the fact that protein sequences for it have remained scarce in databases. The present work circumvented these obstacles by using a combination of shotgun proteomic experiments and different tools to generate and analyze large proteomic datasets and *de novo* sequencing results.

Our results revealed 190 protein identifications, including all classes of toxins described in previous transcriptome analyses^19,20^ (Table 2 (available online only)). Our approach identified both high- and low-abundance toxins of the *L. intermedia* venom, as well as homolog sequences from distinct *Loxosceles* species (astacin-like proteases, PLDs, peptides, TCTPs, hyaluronidases, allergens, serine proteases, serine protease inhibitors, and housekeeping proteins) (Table 2 (available online only)). These data reinforce the holocrine nature of the *Loxosceles* venom gland^23^ and demonstrate that its venom is composed of toxins and housekeeping proteins originating from epithelial-cell content, such as the angiotensin converting enzyme, the 60S ribosomal protein, the Na-Pi co-transporter, and the myosin heavy chain (Table 2 (available online only)). Our results, therefore, validate the method used for analyzing the proteome of an organism with non-sequenced genome.

Taken together, the identified toxins in the *L. intermedia* venom include representatives from all toxin groups, even if in low abundances (as in the case of, e.g., hyaluronidases and serine proteases). We also find it noteworthy that we obtained significant coverage of the three major families present in the venom, viz., PLDs, astacin-like metalloproteases, and ICK peptides. These families are of great importance for studies of the brown-spider envenomation features and of biotechnological and medical applications.

Many of the aligned contigs mapped to distinct PLD isoforms from a variety of *Loxosceles* species. In fact, these toxins are the most studied and well-characterized components of the *Loxosceles* venom^5,20,26,31,46–48^. PLDs are able to reproduce the deleterious effects observed in loxoscelism and represent a great target for drug discovery against brown-spider envenomation^2,5^.

As for the astacin-like metalloproteases identified, we note that astacins were first described as an animal-venom component in 2007 (ref. 28) and only later recognized as a family of toxins present in the *Loxosceles* venom^33^. These toxins present proteolytic activity on distinct extracellular matrix proteins and are related to the hemostatic effects in loxoscelism^43,49^.

ICK peptides, the major components of the *L. intermedia* venom-gland transcriptome (54,9% of the expressed sequence tags), were identified with correspondence to all four different ICK peptides described for *L. intermedia* (LiTx1, LiTx2, LiTx3, and LiTx4)^50,51^. These ICK peptides, also called knottins, are characterized by the neurotoxic properties they exhibit on ion channels and receptors expressed in the nervous systems of insects and mammals^52^. The high expression of LiTx transcripts, which correlates with the proteomic results found herein, are consistent with the venom’s effects of paralyzing and killing both preys and predators^1,20,51^.

## Additional Information

**How to cite this article:** Trevisan-Silva, D. *et al.* A multi-protease, multi-dissociation, bottom-up-to-top-down proteomic view of the *Loxosceles intermedia* venom. *Sci. Data* 4:170090 doi: 10.1038/sdata.2017.90 (2017).

**Publisher’s note:** Springer Nature remains neutral with regard to jurisdictional claims in published maps and institutional affiliations.

## Supplementary Material



## Figures and Tables

**Table 1:** Identifications resulting from the >~10 kDa and <~10 kDa venom fractions

	**>~10 kDa**	**<~10 kDa**	**Together**
**No. of spectral triplets**	41,386	36,625	78,011
**No. of contigs**	642	454	1,096
**No. of homologous sequences**	440	228	190
**No. of** ***de novo*** **sequences**	642	454	1,096
**No. of proteins**	440	228	190
**No. of raw files**	42	46	88
The last column, Together, eliminates redundancies (viz., by maximum parsimony^53^), as well as contaminants and the proteases used during sample preparation (trypsin, chymotrypsin, and pepsin).			

**Table 2:** Meta-contig assemblies output by Meta-SPS on shotgun data from the venom of Loxosceles intermedia and corresponding proteins mapped per similarity

	***de novo*** **sequence**	**Protein index**	**Protein name**	**Species**
Toxin class				
Metalloprotease	[211.812][154.295][261.952]Y[200.869]NMIYGAG	gi 116733934 gb ABK20019.1	astacin-like metalloprotease toxin precursor	*Loxosceles intermedia*
	[168.09]PDATIVY[128.106]	gi 257219865 gb ACV52010.1	astacin-like metalloprotease toxin 2 precursor	*Loxosceles intermedia*
	DDYVI[262.263][128.148]	gi 257219867 gb ACV52011.1	astacin-like metalloprotease toxin 3 precursor	*Loxosceles intermedia*
	GVIIHE[291.835][227.31]GFY	TSA 1, Contig: LIS109	putative astacin-like metalloprotease	*Loxosceles intermedia*
	GTCICDGGSCNC[128.125]R	TSA 1, Contig: LIS137	putative astacin-like metalloprotease	*Loxosceles intermedia*
	[127.832]VRNGCNT[252.829]DVGIR	TSA 1, Contig: LIS187	putative astacin-like metalloprotease	*Loxosceles intermedia*
	RDAP[215.036][170.707][196.097]IG[153.721]F	TSA 2, Contig: LIC247	putative astacin-like metalloprotease	*Loxosceles intermedia*
	GNMFSCWSTVGMR	TSA 2, Contig: LIC331	putative astacin-like metalloprotease	*Loxosceles intermedia*
	INIIY[128.088]	TSA 2, Contig: LIS63	putative astacin-like metalloprotease	*Loxosceles intermedia*
	[128.115]PCGNNV[274.883]DP[227.119]	TSA 3, Contig: LIC321	putative astacin-like metalloprotease	*Loxosceles intermedia*
	GAAGVTIINPAAGR	TSA 3, Contig: LIC369	putative astacin-like metalloprotease	*Loxosceles intermedia*
	NAVSSD[244.341]	TSA 3, Contig: LIS121	putative astacin-like metalloprotease	*Loxosceles intermedia*
	GVIIHEIGHVIGF	sp A0FKN6 VMPA LOXIN	astacin-like metalloprotease toxin precursor	*Loxosceles intermedia*
	[153.983]IDI[128.103][241.226]NTIY	tr C9D7R2 C9D7R2 LOXIN	astacin-like metalloprotease toxin 2 precursor	*Loxosceles intermedia*
	GDSRIWPDGVVIY	tr C9D7R3 C9D7R3 LOXIN	astacin-like metalloprotease toxin 3 precursor	*Loxosceles intermedia*
	F[242.232]D[275.039][128.008]RCVY	TSA 1, Contig: LIC313	putative astacin-like metalloprotease	*Loxosceles intermedia*
	[127.931]D[128.061][241.133]DR[184.194][174.03]PY	TSA 1, Contig: LIC411	putative astacin-like metalloprotease	*Loxosceles intermedia*
	IEAGDV[128.029]GIGCGG	TSA 1, Contig: LIS117	putative astacin-like metalloprotease	*Loxosceles intermedia*
	[349.495]IGITGF	TSA 1, Contig: LIS234	putative astacin-like metalloprotease	*Loxosceles intermedia*
	[184.051]N[257.098][216.857]I[259.978]E[291.043]SIPSN	TSA 1, Contig: LIS98.	putative astacin-like metalloprotease	*Loxosceles intermedia*
	F[127.959]NHDS[204.387]D[242.74]	TSA 2, Contig: LIC228	putative astacin-like metalloprotease	*Loxosceles intermedia*
	RDAP[215.036][170.707][196.097]IG[153.721]F	TSA 2, Contig: LIC247	putative astacin-like metalloprotease	*Loxosceles intermedia*
	GIGIT[128.015]PCTCS[198.07]C[225.217]	TSA 2, Contig: LIC331	putative astacin-like metalloprotease	*Loxosceles intermedia*
	INIIY[128.088]	TSA 2, Contig: LIS63	putative astacin-like metalloprotease	*Loxosceles intermedia*
Phospholipase-D	[198.061]YITAST[234.471]D[127.861][128.324]DFA[128.058]	gi 141452623 gb ABO87656.1	dermonecrotic toxin isoform 6	*Loxosceles intermedia*
	HYEIF[128.043]GFR	gi 156067386 gb ABU43333.1	loxtox i5	*Loxosceles intermedia*
	[241.121]ICAIVI[202.255]G[245.305]II[326.299]	gi 156067390 gb ABU43335.1	loxtox i7	*Loxosceles intermedia*
	[185.016]CC[215.047]DVANAEAW	gi 224472025 gb ACN48894.1	sphingomyelinase D-like protein, partial	*Loxosceles arizonica*
	VATYDDN[283.158]V[248.057][128.175][128.114]	gi 224472109 gb ACN48936.1	sphingomyelinase D-like protein, partial	*Loxosceles spadicea*
	I[128.164][185.033][320.151][199.846]V[128.057]V[257.079]	gi 224472111 gb ACN48937.1	sphingomyelinase D-like protein, partial	*Loxosceles variegata*
	[258.156]GS[215.905]C[128.131]TN[142.105]	gi 224472117 gb ACN48940.1	sphingomyelinase D-like protein, partial	*Loxosceles arizonica*
	[323.223]SIDIIAS[128.107]	gi 224472133 gb ACN48948.1	sphingomyelinase D-like protein, partial	*Loxosceles hirsuta*
	WVI[211.923][323.19][193.88]G[184.835]DWG[288.885]AGVVGGIV[168.053]	gi 224472141 gb ACN48952.1	sphingomyelinase D-like protein, partial	*Loxosceles rufescens*
	IA[277.273]D[217.017]F[286.991]V[275.199]	gi 224472143 gb ACN48953.1	sphingomyelinase D-like protein, partial	*Loxosceles arizonica*
	[127.947]IAEWFDVDVC	gi 224472147 gb ACN48955.1	sphingomyelinase D-like protein, partial	*Loxosceles laeta*
	I[172.141]NFMN[128.005]R	gi 224472157 gb ACN48960.1	sphingomyelinase D-like protein, partial	*Loxosceles laeta*
	AGADGM[212.996]DFP[127.998]	gi 224472195 gb ACN48979.1	sphingomyelinase D-like protein, partial	*Loxosceles aff. Spinulosa GJB-2008*
	[128.05][346.235]FGWEIC[128.103]	gi 224472201 gb ACN48982.1	sphingomyelinase D-like protein, partial	*Loxosceles aff. Spinulosa GJB-2008*
	I[128.005]NYWNNGDNG[230.782]	gi 225008387 gb ACN48920.2	sphingomyelinase D-like protein, partial	*Loxosceles sabina*
	[234.124]IIISI[211.97]T[220.159]Y[128.131]	gi 225008389 gb ACN48924.2	sphingomyelinase D-like protein, partial	*Loxosceles variegata*
	[217.067]VDDGS[127.933][261.153]IGGDSCC[127.973]	gi 225008391 gb ACN48959.2	sphingomyelinase D-like protein, partial	*Loxosceles laeta*
	[202.038]TYEDN[283.238]VTF[128.109]A	gi 49458048 gb AAT66074.1	sphingomyelinase D-like protein 3, partial	*Loxosceles boneti*
	WENF[229.222]FI[128.141]	gi 49458050 gb AAT66075.1	sphingomyelinase D protein 1, partial	*Loxosceles reclusa*
	IATYDDN[283.149][128.13][128.098]	gi 49458052 gb AAT66076.1	sphingomyelinase D protein 2, partial	*Loxosceles reclusa*
	WSR[225.203]IWDIAHM	gi 81343346 gb ABB71184.1	dermonecrotic toxin isoform 3	*Loxosceles intermedia*
	[128.059]SSI[185.218]DN[127.961]AY[128.189]AGVNMATDI[275.181]	gi 90192366 gb ABD91846.1	dermonecrotic toxin isoform 4	*Loxosceles intermedia*
	[259.057]SFADYIDYMR	gi 90192368 gb ABD91847.1	dermonecrotic toxin isoform 5	*Loxosceles intermedia*
	AIC[262.118]N[213.308][157.942]IF[269.463]M	TSA 1, Contig: LIC336	putative phospholipase-D	*Loxosceles intermedia*
	[128.065]PI[257.302][168.016][128.103]	TSA 1, Contig: LIS80	putative phospholipase-D	*Loxosceles intermedia*
	WVI[213.173]FE[230.5]VEDWG[289.028]AGN[198.021]IV[168.053]	TSA 3, Contig: LIC182	putative phospholipase-D	*Loxosceles intermedia*
	CENISTDD[214.057]R	TSA 3, Contig: LIC203	putative phospholipase-D	*Loxosceles intermedia*
	R[169.813][231.411]ANPIGR	TSA 3, Contig: LIC334	putative phospholipase-D	*Loxosceles intermedia*
	[127.988]AE[300.164]D[128.052]IF[128.126]I[255.151]	TSA 3, Contig: LIC352	putative phospholipase-D	*Loxosceles intermedia*
	WVIGG[184.011]A[216.684][200.309]T[200.21][184.253][200.309][216.684]GVVGGIV[168.237]	TSA 3, Contig: LIC395	putative phospholipase-D	*Loxosceles intermedia*
	[255.268]TCEYIEI[245.969]DSNYSEIG[224.144]	TSA 3, Contig: LIC419	putative phospholipase-D	*Loxosceles intermedia*
	GEEYVNVFPMGIR	TSA 3, Contig: LIC423	putative phospholipase-D	*Loxosceles intermedia*
	[256.067][269.811][259.263][227.115]GHEPHC[299.732]	TSA 3, Contig: LIS9	putative phospholipase-D	*Loxosceles intermedia*
	[128.078][128.124]AGV[128.053]D[128.036]EHIW	sp A4USB4 A51 LOXIN	phospholipase D LiSicTox-alphaV1 Dermonecrotic toxin 6	*Loxosceles intermedia*
Toxin class				
Phospholipase-D	[278.133]A[127.914]ARDAG[128.044]V[127.924]	sp B2KKW0 A22 LOXIN	phospholipase D LiSicTox-alphaII2 Loxtox i5	*Loxosceles intermedia*
	TARDVA	sp C0JAQ5 A1IA1 LOXHI	phospholipase D LhSicTox-alphaIA2ai Dermonecrotic toxin	*Loxosceles hirsuta*
	[144.086][196.168][128.016][299.803][217.693]DNGNN[142.216][128.015]	sp C0JAR3 A1IA6 LOXHI	phospholipase D LhSicTox-alphaIA2avi Dermonecrotic toxin	*Loxosceles hirsuta*
	PI[128.15]TD[276.095][260.159][226.968]	sp C0JAR7 A1IA7 LOXHI	phospholipase D LhSicTox-alphaIA2avii Dermonecrotic toxin	*Loxosceles hirsuta*
	[128.069][314.204][266.04]EIIE[128.136]VGY	sp C0JAS6 A1I1 LOXSP	phospholipase D LspaSicTox-alphaIA2i Dermonecrotic toxin	*Loxosceles spadicea*
	[128.135]G[238.403]YEDNPW	sp C0JAT4 A1H1 LOXHI	phospholipase D LhSicTox-alphaIA1i Dermonecrotic toxin	*Loxosceles hirsuta*
	[285.246]TIT[127.957][128.38][193.725]PE[244.902][128.249]F[300.312]	sp C0JAU6 A1LC LOXAR	phospholipase D LarSicTox-alphaIB2c Dermonecrotic toxin	*Loxosceles arizonica*
	IISI[211.095]ID[300.088]	sp C0JAV3 A1KA1 LOXAP	phospholipase D LapSicTox-alphaIB1ai Dermonecrotic toxin	*Loxosceles apachea*
	[128.046]T[261.162][127.9]SAG	sp C0JAX3 A1MA1 LOXDE	phospholipase D LdSicTox-alphaIB3ai Dermonecrotic toxin	*Loxosceles deserta*
	YWTVD[128.227]Y	sp C0JAY0 A1MA6 LOXDE	phospholipase D LdSicTox-alphaIB3ai Dermonecrotic toxin	*Loxosceles deserta*
	GIIISI[250.013]IAHY	sp C0JAZ1 A1OA1 LOXVA	phospholipase D LvSicTox-alphaIC1ai Dermonecrotic toxin	*Loxosceles variegata*
	NAIETDVT	sp C0JAZ4 A1OB1 LOXVA	phospholipase D LvSicTox-alphaIC1bi Dermonecrotic toxin	*Loxosceles variegata*
	[127.882]GI[185.036]EGC[200.002][372.207]ICA	sp C0JAZ8 A1OB5 LOXVA	phospholipase D LvSicTox-alphaIC1bv Dermonecrotic toxin	*Loxosceles variegata*
	R[227.227]TT[154.041]V	sp C0JB02 A1OD LOXRU	phospholipase D LruSicTox-alphaIC1d Dermonecrotic toxin	*Loxosceles rufescens*
	GEND[170.749]N[270.185]AY	sp C0JB04 A1P LOXRU	phospholipase D LruSicTox-alphaIC2 Dermonecrotic toxin	*Loxosceles rufescens*
	[128.095]EVIGVTII[143.89]TCEAH[252.229][210.281]D[274.244]	sp C0JB05 A21 LOXSP	phospholipase D LspaSicTox-alphaII1 Dermonecrotic toxin	*Loxosceles spadicea*
	FCGC[128.12]AWNPGHC[261.261][127.94]	sp C0JB06 A21 LOXVA	phospholipase D LvSicTox-alphaII1 Dermonecrotic toxin	*Loxosceles variegata*
	Y[293.065]PCDCF	sp C0JB07 A21 LOXAP	phospholipase D LapSicTox-alphaII1 Dermonecrotic toxin	*Loxosceles apachea*
	R[312.174][247.035][256.156]AVN	sp C0JB09 A31 LOXAR	phospholipase D LarSicTox-alphaIII1 Dermonecrotic toxin	*Loxosceles arizonica*
	[349.464]NDGCP[314.14]CNDW	sp C0JB12 A332 LOXLA	phospholipase D LlSicTox-alphaIII3ii Dermonecrotic toxin	*Loxosceles laeta*
	N[128.122]AGV[128.154]DREHVW	sp C0JB14 A411 LOXHI	phospholipase D LhSicTox-alphaIV1i Dermonecrotic toxin	*Loxosceles hirsuta*
	VGGSCNDD[127.823]VCC[128.169]GG[128.002]	sp C0JB18 A411 LOXAM	phospholipase D LamSicTox-alphaIV1i Dermonecrotic toxin	*Loxosceles amazonica*
	RIANYDD[345.981][289.109]F	sp C0JB22 A41 LOXAR	phospholipase D LarSicTox-alphaIV1 Dermonecrotic toxin	*Loxosceles arizonica*
	WFDVDVC[128.184]GG	sp C0JB23 A411 LOXLA	phospholipase D LlSicTox-alphaIV1i Dermonecrotic toxin	*Loxosceles laeta*
	[256.225]F[315.247]N[251.495]W[128.071][171.981]GI[214.973]	sp C0JB25 A421 LOXLA	phospholipase D LlSicTox-alphaIV2i Dermonecrotic toxin	*Loxosceles laeta*
	[201.075]DDD[183.982]D[342.088][248.103][282.879]W[260.214]	sp C0JB29 A43 LOXLA	phospholipase D LlSicTox-alphaIV3 Dermonecrotic toxin	*Loxosceles laeta*
	FI[128.05]GDYINV	sp C0JB30 A71 LOXAR	phospholipase D LarSicTox-alphaVII1 Dermonecrotic toxin	*Loxosceles arizonica*
	[127.979]SY[168.058]VIVG[326.398]E[257.168][128.109]D[291.254]	sp C0JB31 A611 LOXHI	phospholipase D LhSicTox-alphaVI1i Dermonecrotic toxin	*Loxosceles hirsuta*
	[243.149]R[214.248]D[228.235]I[128.113][128.353][226.9]TIY	sp C0JB40 B1O LOXCS	phospholipase D LcsSicTox-betaIC1 Dermonecrotic toxin	*Loxosceles cf. spinulosa GJB-2008*
	[249.996]S[320.07]C[292.123]PMI[303.312]V	sp C0JB44 B1T1 LOXSN	phospholipase D LspiSicTox-betaIE3i Dermonecrotic toxin	*Loxosceles spinulosa*
	[260.294]C[274.817]N[250.295]N[229.03][250.931]INNR[248.338]	sp C0JB48 B1S LOXAS	phospholipase D LafSicTox-betaIE2 Dermonecrotic toxin	*Loxosceles cf. spinulosa GJB-2008*
	[128.075]GP[260.085][128.08]FNPGNYDEEE[269.243]	sp C0JB92 B31 LOXSN	phospholipase D LspiSicTox-betaIII1 Dermonecrotic toxin	*Loxosceles spinulosa*
	HGIPCDCGRSCI	sp P0CE78 A1H1 LOXRE	phospholipase D LrSicTox-alphaIA1i Dermonecrotic toxin	*Loxosceles reclusa*
	[185.577]ERR[210.449]WIMG[220.232]	sp P0CE79 A1H2 LOXRE	phospholipase D LrSicTox-alphaIA1ii Dermonecrotic toxin	*Loxosceles reclusa*
	ADNRG[196.202]IW	sp P0CE80 A1HA LOXIN	phospholipase D LiSicTox-alphaIA1a Dermonecrotic toxin 1	*Loxosceles intermedia*
	E[310.079]DRV[128.206][127.978]	sp P0CE83 A1IA1 LOXIN	phospholipase D LiSicTox-alphaIA2ai Dermonecrotic toxin LiP2	*Loxosceles intermedia*
	RN[172.129]A[185.125]IIMAVI	sp Q1KY79 A32 LOXLA	phospholipase D LlSicTox-alphaIII2 Dermonecrotic toxin Ll2	*Loxosceles laeta*
	[128.089]SRD[226.149]DHIW	sp Q1W694 B1Q LOXIN	phospholipase D LiSicTox-betaID1 Dermonecrotic toxin 5	*Loxosceles intermedia*
	[241.168]D[128.065][246.052]D[306.802][314.355]EF	sp Q1W695 A21 LOXIN	phospholipase D LiSicTox-alphaII1 Dermonecrotic toxin 4	*Loxosceles intermedia*
	[144.192][184.287][171.423][244.328][212.328]AFTDD[157.983][314.318]	sp Q27Q54 B1H2 LOXIN	phospholipase D LiSicTox-betaIA1ii Dermonecrotic toxin-like II	*Loxosceles intermedia*
	[271.172]AI[141.933][167.905][170.252]EEI[276.104][127.878]	sp Q2XQ09 B1H1 LOXIN	phospholipase D LiSicTox-betaIA1i Dermonecrotic toxin-like I	*Loxosceles intermedia*
	Y[128.246]E[128.214]IIIF	sp Q5YD77 A1KA LOXBO	phospholipase D LbSicTox-alphaIB1a Dermonecrotic toxin Lb1	*Loxosceles boneti*
	M[169.966]D[227.167]DIA[204.21][238.193]N	sp Q8I912 B1H LOXLA	phospholipase D LlSicTox-betaIA1 Dermonecrotic toxin LlH10	*Loxosceles laeta*
	[252.158][172.24]AG[128.07]IIS[346.153]	sp Q8I913 A331 LOXLA	phospholipase D LlSicTox-alphaIII3i Dermonecrotic toxin LlH13	*Loxosceles laeta*
	I[248.195][172.042]RTNCC[317.124][316.196]	tr G8GZ61 G8GZ61 9ARAC	Sphingomyelinase D A1	*Loxosceles adelaida*
	GMDIPNIRI[198.131][274.115]	TSA 2, Contig: LIS181	putative phospholipase-D	*Loxosceles intermedia*
	IADYE[245.445]RGF	TSA 3, Contig: LIC316	putative phospholipase-D	*Loxosceles intermedia*
	[229.079]VNDYDCA[278.03][197.89][257.788]N[341.945]F[188.387]GGI[173.846]E[203.936][227.312][212.04]	TSA 1, Contig: LIS124	putative phospholipase-D	*Loxosceles intermedia*
ICK-peptide	[288.103][276.948]A[228.135]PETA[217.192]E[258.063]GH[270.214]	gi 118574181 sp Q27Q53.1 TX4 LOXIN	U1-sicaritoxin-Li1c LiTx4	*Loxosceles intermedia*
	[141.867]GCTMGVC[262.034]G[127.99]	gi 74786589 sp Q6B4T3.1 TX3 LOXIN	U2-sicaritoxin-Li1a LiTx3	*Loxosceles intermedia*
	[170.014]T[257.226][128.082]CPAWSHER	gi 74786590 sp Q6B4T4.1 TX2 LOXIN	U1-sicaritoxin-Li1b LiTx2	*Loxosceles intermedia*
	[128.076][198.175]C[128.129][257.053]AWSH[285.151]ECR	gi 74786591 sp Q6B4T5.1 TX1 LOXIN	U1-sicaritoxin-Li1a LiTx1	*Loxosceles intermedia*
	[242.133]VECICSPSYYP[154.122][128.073]	TSA 1, Contig: LIC275	putative LiTx3	*Loxosceles intermedia*
	[128.047]GI[220.146][299.366]THH[128.119]Y[196.17]E[316.185]	TSA 1, Contig: LIC298	putative LiTx3	*Loxosceles intermedia*
	WVI[213.183]FE[230.39][229.002]DWG[288.987][127.905]VV[128.365]V[285.053]	TSA 1, Contig: LIS111	putative LiTx3	*Loxosceles intermedia*
	[174.128]TVPVYAECGR	TSA 1, Contig: LIS14	putative LiTx3	*Loxosceles intermedia*
	[128.108][128.122]NVMRIYVG	TSA 1, Contig: LIS64	putative LiTx3	*Loxosceles intermedia*
	AGGASE[288.32]V[288.068]E	TSA 1, Contig: LIC255	putative LiTx4	*Loxosceles intermedia*
				
Toxin class				
ICK-peptide	[174.137]GIPNASGSIGR	TSA 2, Contig: LIC315	putative LiTx3	*Loxosceles intermedia*
	R[217.187][200.251]FVPVG[174.181]G	TSA 2, Contig: LIS1	putative LiTx3	*Loxosceles intermedia*
	GAD[225.773][283.154][216.215]	TSA 2, Contig: LIS161	putative LiTx3	*Loxosceles intermedia*
	[342.104][142.018][174.19][275.359]TCHGPNWAA[342.104]	TSA 2, Contig: LIS33	putative LiTx3	*Loxosceles intermedia*
	[371.939]WNYA[199.308][128.365]STII	TSA 3, Contig: LIC212	putative LiTx3	*Loxosceles intermedia*
	[128.037]SSFEDF[227.173]VDCNS[261.16]	TSA 3, Contig: LIS21	putative LiTx3	*Loxosceles intermedia*
	G[216.293]CSDG[342.128]DIPC[128.288]	TSA 3, Contig: LIS35	putative LiTx3	*Loxosceles intermedia*
	IGISSD[320.03]PDW[128.125]	TSA 3, Contig: LIS36	putative LiTx3	*Loxosceles intermedia*
	ECI[241.167][241.217][345.95]	TSA 3, Contig: LIS43	putative LiTx3	*Loxosceles intermedia*
	AAGDTN[259.089][229.905][237.813][320.42]W[128.09]	TSA 3, Contig: LIS44	putative LiTx3	*Loxosceles intermedia*
	RCE[224.918]GI[128.1]DISE	TSA 3, Contig: LIS57	putative LiTx3	*Loxosceles intermedia*
	[226.296]PNI[128.014][185.165]T[247.019]CNN[226.997]	TSA 3, Contig: LIC381	putative neurotoxin like-magi-3	*Loxosceles intermedia*
	[258.986]VCYC[208.258]FGV[128.123]NC[128.024]	TSA 3, Contig: LIS20	putative neurotoxin like-magi-3	*Loxosceles intermedia*
	GIRSATTPGNA[128.185]Y	sp P0CE83 A1IA1 LOXIN	phospholipase D LiSicTox-alphaIA2ai Dermonecrotic toxin LiP2	*Loxosceles intermedia*
	[199.081]YD[225.268]DDWCCG[199.016]	sp Q27Q53 TX4 LOXIN	U1-sicaritoxin-Li1c LiTx4	*Loxosceles intermedia*
	[213.19][128.003]Y[127.926][299.318][275.069]R[244.958][183.934][277.077][128.226]G[141.885]	sp Q6B4T3 TX3 LOXIN	U2-sicaritoxin-Li1a LiTx3	*Loxosceles intermedia*
	CT[224.823]CGPYY	sp Q6B4T4 TX2 LOXIN	U1-sicaritoxin-Li1b LiTx2	*Loxosceles intermedia*
	[320.096][128.127][128.065]GTPC[128.107]CPAWSHER[289.113][174.07]	sp Q6B4T5 TX1 LOXIN	U1-sicaritoxin-Li1a LiTx1	*Loxosceles intermedia*
	TG[128.074][128.206]FI	TSA 1, Contig: LIS15	putative LiTx1	*Loxosceles intermedia*
	[260.124][257.99][260.764]DAIESEDPV[208.163]	TSA 1, Contig: LIS159	putative LiTx1	*Loxosceles intermedia*
	SV[127.961][200.118]GI[315.972][128.206]E[142.111]	TSA 1, Contig: LIS163	putative LiTx3	*Loxosceles intermedia*
	M[247.954][271.519]E[198.253][128.081]D[200.22]	TSA 2, Contig: LIS31	putative LiTx3	*Loxosceles intermedia*
	GAG[143.963]NIFA[127.947]	TSA 2, Contig: LIS76	putative LiTx3	*Loxosceles intermedia*
	[127.704]T[128.06]FCV[128.02]NG[128.165]PICP[128.162][167.895]G	TSA 3, Contig: LIC337	putative LiTx1	*Loxosceles intermedia*
	G[266.174]CHAFGSNCR	TSA 2, Contig: LIS229	putative neurotoxin like-magi-3	*Loxosceles intermedia*
Serine protease inhibitor	[128.02]CP[235.787][198.175][143.863]G[241.815]DV[127.896][218.419]G	TSA 1, Contig: LIS209	putative serine protease inhibitor	*Loxosceles intermedia*
Serine protease	[269.335][262.236]S[244.714]WASFP[211.817]SA[128.235]I[269.335]	TSA 1, Contig: LIC305	putative serine protease	*Loxosceles intermedia*
Venom allergen	[346.178][257.117]N[215.219][298.78][217.218]ND[260.144]IG[256.421][372.04]	TSA 2, Contig: LIC179	putative venom allergen	*Loxosceles intermedia*
Hyaluronidase	V[207.937]SSEY[211.831]I[326.363][319.484]	TSA 1, Contig: LIS222	putative hyaluronidase	*Loxosceles intermedia*
TCTP	[256.849][275.401][210.175]I[252.242]TA[244.108][200.309][241.062]N[170.078]IIED[372.344]	tr G3LU44 G3LU44 LOXIN	translationally-controlled tumor protein homolog LiTCTP	*Loxosceles intermedia*
	[269.086]FGIMAP[225.403]GEIR	gi 344995179 gb AEN55462.1	translationally controlled tumor protein LiTCTP	*Loxosceles intermedia*
**Cellular processes proteins**	[200.059]VHEDNI[128.065]E[128.062]H[128.193][262.127]	TSA 1, Contig: LIC216	putative 60S ribosomal protein L10	*Loxosceles intermedia*
	[199.078]CT[297.052]F[264.084][233.709]GCPSR	TSA 1, Contig: LIS275	putative cysteine-rich PDZ-binding protein	*Loxosceles intermedia*
	[269.045]D[173.858]DPDCDC[211.944]D[173.937]D[269.044]	TSA 1 Contig: LIS239	putative cytochrome P450 mRNA	*Loxosceles intermedia*
	I[204.06]CV[128.097][297.04]	TSA 1, Contig: LIS199	putative histone H2B	*Loxosceles intermedia*
	[241.241][210.11]DFYEI[170.035]SA[262.036]	TSA 1, Contig: LIS189	putative myosin heavy chain	*Loxosceles intermedia*
	[128.159]GF[218.024]D[127.978]VVIA	TSA 1, Contig: LIC349	putative secreted salivary gland peptide	*Loxosceles intermedia*
	[262.073]I[243.142]PSNPSCR	TSA 2, Contig: LIC232	putative 60S ribosomal protein L27	*Loxosceles intermedia*
	[216.109]IVISNPDINH[260.307][226.167][128.074]	TSA 2, Contig: LIC283	putative actin related protein 3	*Loxosceles intermedia*
	TED[128.098]V[229.139]	TSA 2, Contig: LIS258	putative glycoprotein hormone alpha-2 precurso	*Loxosceles intermedia*
	[128.037]SSFEDF[227.173]VDCNS[261.16]	TSA 2, Contig: LIS217	putative myosin light chain	*Loxosceles intermedia*
	[199.18]SIDIIAS[128.129]DVMDR	TSA 2, Contig: LIS39	putative Na/Pi co-transporter	*Loxosceles intermedia*
	[269.086]FGIMAP[225.403]GEIR	TSA 2, Contig: LIS280	putative ribosomal protein L18a	*Loxosceles intermedia*
	[247.11]G[128.088]I[183.991]N[210.738][262.174][285.2]	TSA 2, Contig: LIS213	putative ribosomal protein S3	*Loxosceles intermedia*
	Y[127.918]GV[143.679]I[196.241]AGR	TSA 3, Contig: LIC307	putative angiotensin-converting enzyme	*Loxosceles intermedia*
	VGSIPIDI[128.058]	TSA 3, Contig: LIC368	putative myosin light chain	*Loxosceles intermedia*
	[128.071]SAI[250.284]IS[127.926]N	TSA 3, Contig: LIC207	putative troponin C	*Loxosceles intermedia*
	GTIPVT[323.12]P[128.197]	TSA 3, Contig: LIS253	putative troponin T	*Loxosceles intermedia*
	[128.057]CW[283.922]E[127.782][128.342]GA	TSA 3, Contig: LIS216	putative zinc finger protein	*Loxosceles intermedia*
	[128.066]CG[169.924]S[293.695]D[211.062]C[143.962]	TSA 1, Contig: LIS257	putative cAMP-responsive element-binding protein-like 2-like	*Loxosceles intermedia*
	HVSV[128.009]	TSA 1, Contig: LIC219	putative glutamine synthetase	*Loxosceles intermedia*
	[262.165]P[230.085]D[128.062]	TSA 2, Contig: LIS220	putative 60S ribosomal protein L6	*Loxosceles intermedia*
	[268.215]HCTCDDVC[128.023]R[275.259]Y	TSA 2, Contig: LIS231	putative calmodulin	*Loxosceles intermedia*
	[274.04]TPIRIN[210.228]II[228.096]	TSA 2, Contig: LIS27	putative Na/Pi co-transporter	*Loxosceles intermedia*
	[372.255]DEIIN[171.15]IE[200.054]E[201.127]S[226.48]HG[293.174][372.288]	TSA 2, Contig: LIS265	putative selenoprotein M	*Loxosceles intermedia*
	NW[211.918]RIR[298.994][248.176]	TSA 2, Contig: LIS205	putative ubiquitin	*Loxosceles intermedia*
	[253.085]IG[200.301]T[251.496][171.357]ED	tr Q6W974 Q6W974 LOXRE	codium/potassium ATPase alpha subunit	*Loxosceles reclusa*
	YHNN[232.454]ISII	tr B8R316 B8R316 9ARAC	NADH-ubiquinone oxidoreductase chain 1	*Loxosceles baja*
	[276.09]MISMEVG[307.07][259.494]Y	tr B8R320 B8R320 LOXDE	NADH-ubiquinone oxidoreductase chain 2	*Loxosceles deserta*
Cellular processes proteins				
	[128.205]MFTIIM[235.906]GV[215.128]	tr B8R326 B8R326 9ARAC	NADH-ubiquinone oxidoreductase chain 1	*Loxosceles chinateca*
	[245.288]RA[260.078]NTSTIGTA[212.046]R	tr B8R332 B8R332 9ARAC	NADH-ubiquinone oxidoreductase chain 1	*Loxosceles kaiba*
	S[246.297][301.019]IVS[170.255]H	tr B8R341 B8R341 9ARAC	NADH-ubiquinone oxidoreductase chain 1	*Sicarius aff. patagonicus*
	RT[255.953][231.083][299.111]CVG	tr C1ITN3 C1ITN3 LOXAS	cytochrome c oxidase subunit 1	*Loxosceles aff. spinulosa*
	I[211.14][268.32]I[187.685]IV[246.077]	tr C1ITP7 C1ITP7 9ARAC	cytochrome c oxidase subunit 1	*Sicarius dolichocephalus*
	FF[238.18]IITAG[273.235]	tr C1ITR2 C1ITR2 9ARAC	cytochrome c oxidase subunit 1	*Drymusa serrana*
	YEDRIVVR[230.004]	tr C1ITR3 C1ITR3 9ARAC	cytochrome c oxidase subunit 1	*Drymusa dinora*
	[316.142]TVYCMSIEITA[226.21]IEDI[241.221]	tr C1IZB2 C1IZB2 LOXLA	cytochrome c oxidase subunit 1	*Loxosceles laeta*
	GVI[248.157]NITGYR	tr C5J3X9 C5J3X9 LOXRU	cytochrome c oxidase subunit 1	*Loxosceles rufescens*
	IA[128.08]MFTIIE[216.039]GV[215.166]	tr B8R317 B8R317 LOXAP	NADH-ubiquinone oxidoreductase chain 1	*Loxosceles apachea*
	[278.338]A[234.386]WHVEN[210.973]VNI[210.062]	tr B8R335 B8R335 LOXS4	NADH-ubiquinone oxidoreductase chain 1	*Loxosceles sp.*
	[265.105]V[213.051][172.098]A[283.126]Y[201.071][210.087][278.104][269.011]RGIVMV[283.117]	tr C1ITR3 C1ITR3 9ARAC	cytochrome c oxidase subunit 1	*Drymusa dinora*
	[127.983]AG[153.948]ACTGEMGSCG	tr C5J3X7 C5J3X7 LOXGA	cytochrome c oxidase subunit 1	*Loxosceles gaucho*
	YSVFCMM[128.12]V	tr C5J3X9 C5J3X9 LOXRU	cytochrome c oxidase subunit 1	*Loxosceles rufescens*
	[225.309]SWD[217.039][271.219]C[258.226][218.143][302.82]GMIGSEN	tr Q6W974 Q6W974 LOXRE	sodium/potassium ATPase alpha subunit	*Loxosceles reclusa*
The contigs are listed according to their toxin class.				

## References

[d1] Trevisan-Silva D. 2016 Dummy PXD005523

[d2] Figshare Trevisan-Silva D. 2017 https://doi.org/10.6084/m9.figshare.c.3709168

